# Selection of a Bacterial Conditioner to Improve Wheat Production Under Salinity Stress

**DOI:** 10.3390/microorganisms13102273

**Published:** 2025-09-28

**Authors:** Ramila Fares, Abdelhamid Khabtane, Noreddine Kacem Chaouche, Miyada Ouanes, Beatrice Farda, Rihab Djebaili, Marika Pellegrini

**Affiliations:** 1 Laboratory Biotechnology, Water, Environment and Health, Abbes Laghrour University, Khenchela 40004, Algeria; ramila.fares@graduate.univaq.it (R.F.);; 2 Department of Life, Health and Environmental Sciences, University of L’Aquila, 67100 L’Aquila, Italy; beatrice.farda@graduate.univaq.it (B.F.); rihab.djebaili@univaq.it (R.D.); 3 Laboratory of Mycology, Biotechnology and Microbial Activity, University of Constantine 1, Brothers Mentouri, Constantine 25017, Algeria; noreddine.kacem.chaouche@umc.edu.dz

**Keywords:** bacterial inoculant, durum wheat, plant growth promoter, salt stress, sustainability

## Abstract

This study investigated the isolation and formulation of a bacterial conditioner as a biostimulant for *Triticum durum* (durum wheat) under salinity stress. An Algerian alkaline–saline soil was sampled, characterized for its physical and chemical characteristics and its culturable and total microbial community (16S rRNA gene metabarcoding). Three bacterial strains showing high 16S rRNA gene similarity to *Pseudomonas putida*, *Bacillus proteolyticus*, and *Niallia nealsonii* were selected for their plant growth-promoting (PGP) traits under different salinity levels, including phosphate solubilisation (194 µg mL^−1^), hormone production (e.g., gibberellin up to 56 µg mL^−1^), and good levels of hydrocyanic acid, ammonia, and siderophores. *N. nealsonii* maintained high indole production under saline conditions, while *B. proteolyticus* displayed enhanced indole synthesis at higher salt concentrations. Siderophore production remained stable for *P. putida* and *N. nealsonii*, whereas for *B. proteolyticus* a complete inhibition was registered in the presence of salt stress. The consortium density and application were tested under controlled conditions using *Medicago sativa* as a model plant. The effective biostimulant formulation was tested on *Triticum durum* under greenhouse experiments. Bacterial inoculation significantly improved plant growth in the presence of salt stress. Root length increased by 91% at 250 mM NaCl. Shoot length was enhanced by 112% at 500 mM NaCl. Total chlorophyll content increased by 208% at 250 mM NaCl. The chlorophyll a/b ratio increased by 117% at 500 mM. Also, reduced amounts of plant extracts were necessary to scavenge 50% of radicals (−22% at 250 mM compared to the 0 mM control). Proline content increased by 20% at both 250 mM and 500 mM NaCl. These results demonstrate the potential of beneficial bacteria as biostimulants to mitigate salt stress and enhance plant yield in saline soils.

## 1. Introduction

*Triticum durum* Desf., known as durum wheat, is a staple crop cultivated globally on 17 million hectares of land [1]. This crop has a great relevance in global agriculture and food production, and over 38 million tons are yielded annually [2]. Algeria is one of the primary cereal-producing countries, with 17% of its area under cultivation, nearly 9% in the lower lands and sub-Saharan agricultural regions, and 90% of the cultivation is with durum wheat. Climate changes challenge the Algerian *T. durum* annual yield. Since 2020, its cultivations have increased [3].

In the semi-arid regions of Algeria, the yield and productivity of durum wheat face significant challenges from both biotic and abiotic stress factors, such as salinity [4,5]. Salt stress is becoming the most important stress factor in terms of land losses, climate change, and limited water availability in cultivated land [6]. Salinity stress is associated with 25% of the total irrigated land, causing 15% of land loss in the world. There are also regions where it causes crop production losses ranging from 70% to 100% of their potential yields [7].

Depending on the geography and pedo-climatic factors, the elements causing salinization of the soil change, and salinity can be associated with chlorinity or sodicity. These salts have several negative effects on plant cellular wall osmotic potential and growth [8]. Salinity stimulates the production of reactive oxygen species (ROS), which leads to oxidative stress. Growth reduction during the phase of seedling emergence is one of the first described salt-induced damages. Furthermore, the metabolic photosynthesis rate and activity of broad-leaved plant leaves decrease [9]. Soil salinization affects plant water uptake due to the generation of a strong osmotic potential in plant cells, creating contradictory pressure [10]. Salinity inhibits cell division, but as the osmotic potential increases, cell expansion occurs. Several compounds flow from the root of the plant to increase the water absorption rate quickly. Many of them are hormones, especially auxins. The Na^+^ accumulation in the chloroplasts and its uptake from the leaves causes chlorophyll degradation [11].

Salinization can be restored by chemical remediation (e.g., leaching, rinsing, gypsum, and lime treatment). However, these treatments are time-consuming and reduce plant and microbial biodiversity [12]. Recently, the use of halotolerant bacteria has been proposed as a valid and environmentally friendly tool to restore degraded saline lands and induce plant halotolerance [13]. Inoculating plants with salt-tolerant bacteria (STB) under salinity stress has been shown to induce significant physiological and biochemical adjustments that enhance stress resilience. These bacteria promote osmotic balance through the accumulation of osmoprotectants such as proline and exopolysaccharides (EPS), while also stimulating antioxidant defense systems to alleviate oxidative damage. Moreover, STB facilitate nutrient acquisition and contribute to lowering Na^+^/K^+^ ratios in plant tissues, thereby mitigating the toxic effects of excessive sodium. Collectively, these mechanisms sustain cellular homeostasis, improve plant growth, and enhance productivity under saline conditions [14]. Plant–microbe interactions are crucial for maintaining soil health and enhancing plant adaptation to various environmental stresses. Many reports on beneficial bacteria demonstrate that these bacteria can enhance plant growth, increase crop yield, and facilitate nutrient mobilization. By using nitrogen fixation, phosphate solubilization, and siderophore synthesis, these microorganisms are utilized as biofertilizers and agricultural biocontrol agents to increase crop resilience to a variety of challenges [15,16]. Furthermore, PGPR contributes to the regulation of plant hormones, particularly through the production of growth-promoting hormones (auxin and cytokinin), the reduction in ethylene concentration through the action of ACC deaminase (ACC lyase) on the degradation of ACC (ethylene precursor), and the enhancement of plant stress tolerance [17].

Although several studies have demonstrated the beneficial effects of salt-tolerant bacteria on plant growth, most of them have focused on a limited number of crops and under moderate salinity conditions. The combined evaluation of their impact on both wheat and alfalfa under severe salinity remains insufficiently addressed [18]. With this work, we hypothesised that Algerian agricultural soil used for wheat cultivation could be a valuable resource of beneficial bacteria with salt stress tolerance-inducing activity. Therefore, this research aimed at selecting a bacterial conditioner to improve wheat production under salinity stress. We isolated bacteria from the rhizosphere of durum wheat and characterized the strains for their plant growth-promoting traits under saline conditions. Consortia were developed with the three best strains and tested on *Medicago sativa* under controlled conditions. The best formulation was tested on a salt-sensitive durum wheat cultivar under greenhouse.

## 2. Materials and Methods

### 2.1. Soil Sampling

Soil was sampled from a field of saline-tolerant durum wheat grown by Cosider Agricole at Mita Babar in the Khenchela region of Algeria (34°36′21″ N, 7°13′09″ E). The soil was sampled following a systematic x-pattern. The sampling area of 1 hectare was divided into imaginary units, and following an x-path, three sub-samples were taken within each unit, avoiding sampling in field edge areas or anomalous areas of negligible importance to the plot (e.g., sinks, stagnation areas). Sub-samples were collected on a cloth and homogenised to form a single 2 kg bulk sample. The bulk sample was divided into aliquots and stored at −80 °C and room temperature for further analysis.

#### 2.1.1. Soil Physical-Chemical Profile

In accordance with the Italian “Ministero Delle Politiche Agricole Alimentari e Forestali” regulations, soil samples that had been dried at room temperature, crushed, and sieved to remove particles larger than 2 mm were subjected to physical-chemical analysis [19].

#### 2.1.2. Microbial Community Metabarcoding

Soil Microbial community metabarcoding analysis was performed on soil samples stored at −80 °C. Before DNA extraction, five aliquots were thawed and pre-incubated at room temperature for 1 h. Aliquots were extracted using the NucleoSpin Soil kit (Macherey Nagel, Düren, Germany), following the manufacturer’s instructions. DNA concentration was measured using a Nanodrop 2000 (Thermo Scientific, Waltham, MA, USA) and verified on 2% agarose gels. Aliquots were subsequently combined into equimolar mixtures and sent for external purification and sequencing at BMR Genomics (Padua, Italy). The previously published sequencing and bioinformatic approach was followed [20].

### 2.2. Bacterial Isolation

The serial-dilution technique was used to isolate cultivable microorganisms. After dissolving 10 g of dirt in 90 mL of sterile physiological water (0.9%), the mixture was rapidly agitated for the whole night. A series of decimal dilutions (up to 10^−6^) were performed in conjunction with this process. 100 µL of each dilution were plated onto Luria–Bertani (LB) and King B (KB) agar media (Liofilchem, Roseto degli Abruzzi, Italy). The resulting colonies were isolated and put through morphological and biochemical evaluation after these cultures were incubated on plates at 28 °C for 48 h. Following purification, the strains were kept at −80 °C for storage [21].

### 2.3. In Vitro Plant Growth-Promoting Traits

In vitro plant growth-promoting traits were characterised following the approaches previously described [20].

For Phosphate estimation, bacterial isolates were inoculated to NBRIP (National Botanical Research Institute’s Phosphate Growth Medium) broth with tricalcium phosphate Ca_3_(PO_4_)_2_ at 30 °C for 7 days with moderate agitation according to [22]. Grown cultures were centrifuged for 15 min at 700× *g* and 0.1 mL of SnCl_2_ and 1 mL of ammonium molybdate were combined with 0.1 mL of supernatant. The spectrophotometric determination was carried out at 600 nm according to the Olsen and Sommers method [23]. Results were expressed as µg PO_4_^3−^ mL^−1^.

TSB medium supplemented with 0.2% L-tryptophan was used to quantify indoles. The cultures were centrifuged for 10 min at 8000× *g* to identify the synthesis of indole. After that, 4 mL of Salkowski reagent was combined with 1 mL of supernatant, and the combination was left to incubate for 30 min in the dark. The development of a pink hue signified the formation of indole. The wavelength at which the optical density was measured was 530 nm. The results were calculated using the standard calibration curve and given in µg of IAA equivalents mL^−1^.

To quantify gibberellin (GB) content, LB medium supplemented with 1% L-tryptophan was incubated at 30 °C with moderate agitation. For fifteen minutes, the culture was centrifuged at 1600× *g*. After that, two mL of zinc acetate were added to the supernatant, and it was incubated for two minutes. Two mL of potassium ferrocyanide were then added, and the mixture was centrifuged for fifteen minutes at 300× *g*. Five mL of 30% HCl were added for every five mL of supernatant. After that, the samples were incubated for 75 min at room temperature. At 254 nm, the absorbance was measured. Gibberellic acid (Sigma, St. Louis, MO, USA) was used as reference standard (y = 0.49x + 0.07; R^2^ = 0.913) and the results were expressed as µg GB mL^−1^ [24].

The ACC deaminase activity was performed as described by [25]. In brief, 15 mL of liquid LB medium were inoculated with 200 μL of bacterial suspension and incubated for 3 days at 30 °C with moderate agitation. After centrifugation, the cultures were washed, and the cell pellets were suspended in 15 mL of minimal DF medium containing 3 mM ACC and incubated for 3 days at 30 °C with shaking. The bacterial suspensions were then centrifuged and the pellets obtained were used for the determination of enzyme activity. The pellets were resuspended in 400 µL of 0.1 M Tris HCl buffer pH 8.0 with 20 µL of toluene. 50 µL of the cell-free lysate was divided into 3 microtubes, 2 of which were supplemented with 5 µL of ACC (0.5 M). The third served as a negative control. Another negative control was performed by adding 50 µL of 0.1 M Tris HCl pH 8.0 and 5 µL of ACC (0.5 M). The samples were vortexed for 5 s and then incubated at 30 °C for 30 min. At the end of the incubation period, each tube was treated with 500 µL of 0.56 M HCl, then the tubes were vortexed, and the samples were centrifuged at 10,000× *g* for 5 min. The absorbance of the reaction mixtures was measured at 540 nm using a Cary Bio 50 UV-Vis spectrophotometer (Agilent, Santa Clara, CA, USA). ACC deaminase activity was determined from a calibration curve of α-ketobutyrate (5, 10, 15, 20, and 25 µmol mL^1^) and expressed in µmol of α-ketobutyrate per hour per mg of protein.

To identify the most promising microbial strains based on plant growth-promoting (PGP) traits, a multi-criterion ranking analysis was performed. The three quantitative parameters, phosphate solubilization, gibberellic acid production, and indole production, were considered. Each strain was independently ranked for each trait in descending order, assigning a rank of 1 to the highest value within a trait. Ties were assigned the minimum rank shared by the tied values. For each strain, a cumulative score was then calculated as the sum of the individual ranks across the three traits. Strains with the lowest total scores were considered the most effective overall, reflecting consistently high performance across evaluated PGP traits.

Selected strains were investigated again for phosphate solubilization, gibberellic acid production, and indole production in the presence of salt stress. Siderophore, ammonia, and hydrocyanic acid productions were also screened in the absence and presence of salt stress as previously described [20].

### 2.4. 16S rRNA Barcoding and Phylogenetic Study

The most intriguing PGP strains selected based on their PGP traits under salinity stress were molecularly analysed using 16S barcoding (BMR Genomics, Padua, Italy). DNA was amplified by direct PCR using universal bacterial primers (27F/1492R) and then sequenced. The obtained sequence (~1400 bp) was compared with those in the NCBI genetic database using the BLAST 2.17.0 tool (National Center for Biotechnology Information; http://www.ncbi.nlm.nih.gov/; accessed on 3 May 2025). A sequence identity rate of more than 99% was used. The maximum likelihood method was used to infer the tree applying the model that obtained the lowest Bayesian Information Criterion value. Analyses were conducted in MEGA11 [26].

### 2.5. Consortium Formulation

Strains showing good in vitro PGP activities were selected, and a compatibility test was performed between the strains chosen using the cross-streak method. Bacterial cultures were inoculated onto the same culture medium and incubated at 28 ± 1 °C for 48 to 72 h. The absence of inhibition zones around the colonies indicates the compatibility of the strains and their mutual non-antagonism. Selected isolates were grown in 250 mL flasks containing 100 mL of TSB culture medium (NutriSelect Plus, Merck, San Jose, CA, USA). The cultures were incubated under moderate agitation (New Brunswick Scientific Co., Inc., Edison, NJ, USA) to provide optimum conditions for their growth at 48 h.

### 2.6. Controlled Condition Experiment on Medicago sativa

Alfalfa seeds were surface sterilised by immersing them in a 20% sodium hypochlorite solution and then rinsed three times with sterile distilled water. After that, the seeds were put in 70% ethanol for 1 min, followed by three rinses with sterile distilled water. The seeds were inoculated by immersing them in bacterial suspensions adjusted to 10^6^, 10^7^, and 10^8^ CFU mL^−1^ for two hours. Alfalfa was then cultivated under salinity stress in the presence and absence of the inoculum by mixing the soil with different NaCl concentrations as presented in Table 1. Three seeds were sown in pots of a diameter of 10 cm, a height of 12 cm, and a soil capacity of 900 g. The interiors of the pots were disinfected using 70% ethanol. The plants were grown under natural light and were watered with sterile tap water for 10 days. Each experimental unit consisted of three replications [27]. Germination was monitored, and after 40 days after sowing (DAS), the roots and shoots were harvested separately, and their lengths were measured. Additionally, the chlorophylls were measured as previously described [28].

### 2.7. Greenhouse Experiment on Triticum durum

The durum wheat seeds were inoculated by immersing them in bacterial suspensions adjusted to 10^6^ CFU mL^−1^ (using MacFarland standard method) for two hours (best condition on alfalfa) without sterilisation. The control was treated with tap water. The salinity stress was applied by mixing the soil with different NaCl concentrations (from 0 to 1.5 mol L^−1^). The experiment was performed as follows:

- A, no bacteria and no salt stress
- B, no bacteria and salt stress 250 mM
- C, no bacteria and salt stress 500 mM
- D, no bacteria and salt stress 750 mM
- E, no bacteria and salt stress 1500 mM
- F, bacteria and no salt stress
- G, bacteria and salt stress 250 mM
- H, bacteria and salt stress 500 mM
- I, bacteria and salt stress 750 mM
- J, bacteria and salt stress 1500 mM

Both inoculated and non-inoculated seeds were sown in pots (diameter of 10 cm) containing three seeds each. The interiors of the pots were disinfected using 70% ethanol. The plants were grown under natural light and were watered with tap water for 10 days. Each experimental unit consisted of three replications. Forty days after sowing, the roots and shoots were harvested separately, and their lengths were measured. Additionally, the fresh weight (FW) and dry weight (after being oven-dried at 105 °C for 48 h) were recorded [28].

### 2.8. Plant Analyses

#### 2.8.1. Chlorophyll Evaluation

Chlorophylls (i.e., chlorophyll a (Chl_a_) + chlorophyll b (Chl_b_)) concentration was assessed following the method described by [29], 0.5 g of leaf or stem tissue from each sample were cut and homogenised in 10 mL of 80% acetone before being kept at 10 °C overnight in the dark, following centrifugation at 15,000× *g* for 5 min. The absorbance of the supernatant was assessed at 663 and 645 nm using a Multiskan GO Microplate Spectrophotometer (Thermo Scientific, Cleveland, OH, USA). The following equations were used to estimate chlorophylls. The results were presented as mg 100 g FW^−1^.

(1) $${Ch}_{a}\;(\mathrm{mg}\;L^{-1})=12.41\;(\mathrm{OD}\;663)-2.59\;(\mathrm{OD}\;645)$$

(2) $${Ch}_{b}\;(\mathrm{mg}\;L^{-1})=22.9\;(\mathrm{OD}\;645)-4.68\;(\mathrm{OD}\;663)$$

(3) $${Ch}_{tot}\;(\mathrm{mg}\;L^{-1})=\mathrm{Cha}+\mathrm{Chb}$$

#### 2.8.2. Estimation of the Proline Content

Using the method outlined by [30], the content of proline in fresh leaves was determined 40 days after sowing. Five mL of a 60:25:15 methanol: chloroform: distilled water combination was added to tubes containing 0.5 g of leaves. For two minutes, the tubes were heated to 60 °C. For ten minutes, the mixtures were centrifuged at 8000× *g*. Four mL of Ninhydrin solution, four mL of glacial acetic acid, and one mL of distilled water were combined with the supernatant. Prior to measuring the absorbance at 520 nm, the mixture was heated to 90 °C for 45 min and then allowed to cool to room temperature. Proline was used as a reference standard (y = 0.0314x + 0.0409; R^2^ = 0.9993) to express results in g proline per g fresh weight (g Pro g FW^−1^).

#### 2.8.3. Evaluation of Antioxidant Activity

The DPPH (1,1-diphenyl-2-picryhydrazyl, Sigma-Aldrich, St. Louis, MO, USA) radical scavenging capability was used to measure the antioxidant activity. 1.6 mL of a 0.002% methanolic DPPH solution was combined with 2 mL of each plant extract concentration (50 mg mL^−1^). For half an hour, the mixture was incubated at room temperature in the dark. At a wavelength of 517 nm, the absorbance was measured against a blank, and ascorbic acid served as a positive control. The findings were presented as IC50 values, which indicate the concentration needed to attain 50% inhibition (µg mL^−1^) of DPPH free radicals [31,32].

### 2.9. Statistical Analysis

All data collected represent the means of three replicates and data were reported as means ± standard deviation. Data were tested for normality using the Jarque–Bera test and analyzed using two-way analysis of variance (ANOVA) and Fisher LSD post hoc for the comparison of the means (α = 0.001). All statistical calculations were performed using XLSTAT 2016 (Addinsoft, Paris, France).

## 3. Results

### 3.1. Soil Physical-Chemical Profile

The soil had the following characteristics: EC 451 µS/cm, pH 7.98, CaCO_3_ 16%, organic carbon 0.98%, organic matter 17%, nitrogen levels 0.1%, soil moisture 72%, particle size distribution 28% silt, clay 29%, and sand 44%. With a moderately high electrical conductivity of 450.53 µS/cm, there is some salinity present, enough to harm salt-sensitive crops. The pH sits at 7.98, indicating an alkaline environment, which, combined with the high calcium carbonate content (15.84%), points to calcareous soil-common in arid regions and known for locking up certain micronutrients like iron and phosphorus. Organic matter and organic carbon were present in moderate amounts (1.69% and 0.98%, respectively) suggesting sufficient biological activity. However, the nitrogen level was relatively low (0.084%) limiting plant growth without supplementation. The soil is very moist at 71.81%, which could either be beneficial or problematic depending on drainage conditions. Texturally, it leans toward a loam with 44% sand, 28% silt, and 29% clay, offering a good balance for root development and water movement.

### 3.2. Microbial Community Metabarcoding

As presented in Table 2, the bacterial community profile revealed through 16S rRNA gene metabarcoding, showed a diverse and well-balanced microbial community.

**Figure 1:**
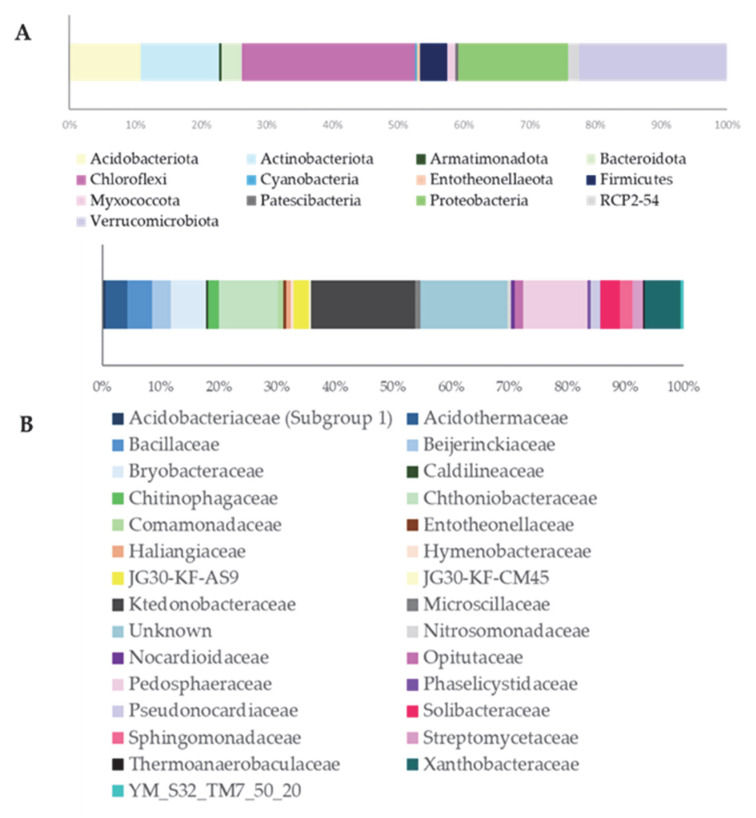
ASVs distribution at the Phylum (**A**) and Family (**B**) level.

With 77 observed taxa and nearly 15,000 sequence reads, the community exhibits high richness and evenness, supported by a low dominance index (D = 0.01985) and strong diversity metrics (Simpson 1-D = 0.98; Shannon H = 4.10). As shown in Figure 1A, at the phylum level, Chloroflexi dominates, accounting for over 1/4 of the total Amplicon Sequence Variants (ASVs), followed by Proteobacteria, Verrucomicrobiota, Actinobacteriota, and Acidobacteriota. At the family level (Figure 1B, Table S1), Ktedonobacteraceae stands out, reflecting the prominence of Chloroflexi.

**Figure 2:**
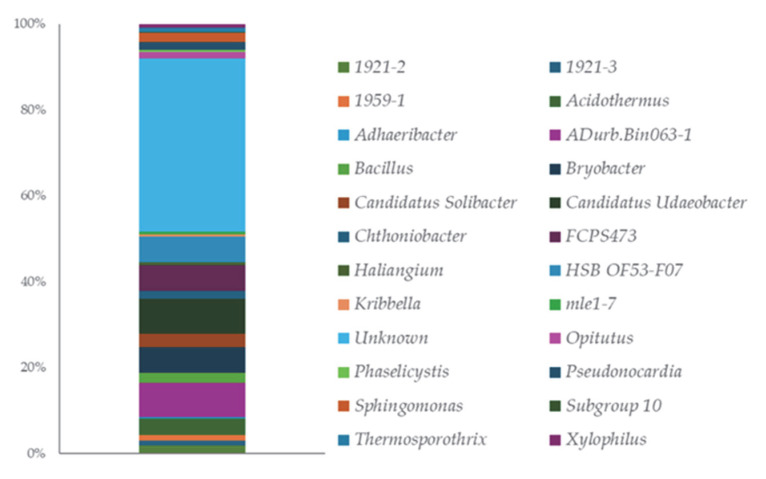
ASVs distribution at the Genus level.

Genus-level distribution (Figure 2, Table S2) reveals a striking presence of uncultured or provisionally named taxa (e.g., 1921-2 and 1959-1), pointing to an underexplored microbial landscape. The presence of genera with key activities in plant growth promotion can be observed, including *Bacillus*, *Pseudonocardia*, and *Sphingomonas*.

### 3.3. In Vitro Plant Growth-Promoting Traits

**Figure 3:**
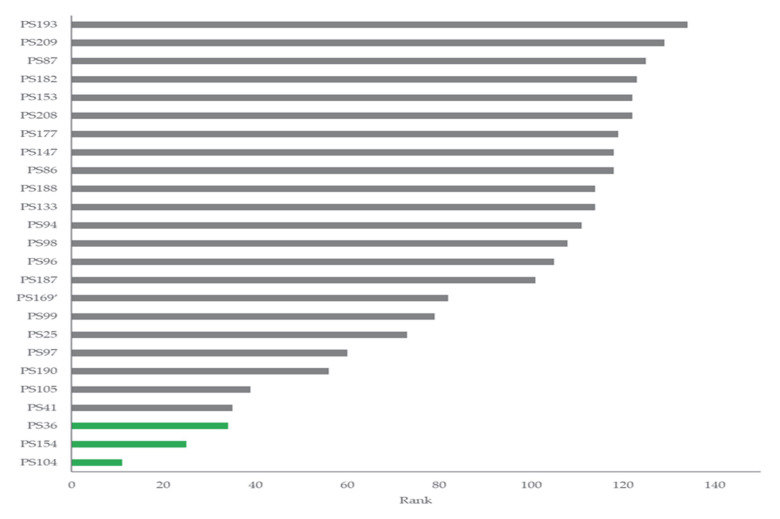
Ranking analysis showing the best-performing 25 strains.

Figure 3 and Table S4 present the results of the PGP screening on 134 isolates.

**Figure 4:**
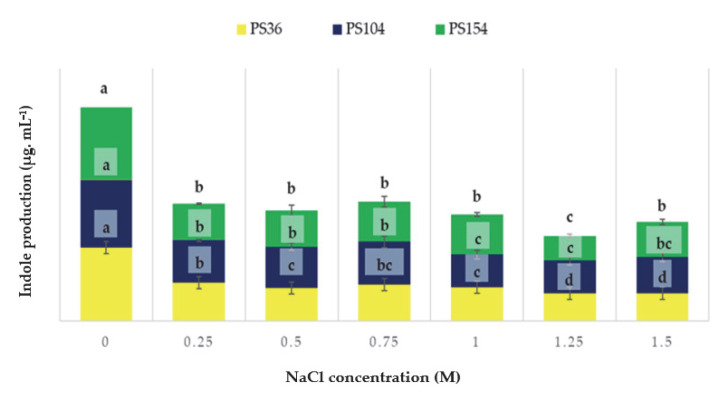
Indole production by the three selected strains in the presence of salt stress. For the same strains, results followed by the same letter are not statistically significant according to the Fisher LSD post hoc test (*p* < 0.05). Least significant differences: PS36, 3.86; PS104, 7.67; PS154, 6.67.

All the strains exhibited positive phosphate solubilization and produced gibberellic acid and indoles. However, the presence of good results for these three key PGP traits was observed only for 10 strains (total score < 100 in Table S4). Based on ranking analysis (Figure 3, Table S4), strains PS36, PS104, and PS154 were selected and screened for PGP traits under salinity stress. Even if a general decrease of 3–6% was recorded for phosphate solubilisation and gibberellic acid production with increasing salinity stress application, the comparison of the means showed no statistical significance among treatments. The phosphate solubilization average values for strains PS36, PS104, and PS154 were 194.13 ± 4.41; 93.11 ± 1.61; and 147.49 ± 1.88, respectively. The gibberellic acid production average values for strains PS36, PS104, and PS154 were 21.51 ± 1.36; 56.41 ± 2.33; 31.52 ± 2.47, respectively. Figure 4 shows the concentration of indole produced by the different strains with and without salt concentrations.

The obtained results revealed that salinity significantly affected indole production for all the strains tested. Strain PS154 maintained significant indole production even at high NaCl concentrations. Conversely, other strains showed a progressive decline starting from 1 M. As reported in Table 3, a different behaviour was shown for HCN, NH_3_, and siderophores production.

In the absence of salt, high levels of HCN production were observed. Strain PS104 exhibited moderate HCN production. When the strains were exposed to NaCl, the production of HCN varied significantly. PS36 demonstrated a remarkable salt tolerance, maintaining high levels of HCN production even at a concentration of 0.75 mol L^−1^ NaCl. At 1 and 1.25 mol L^−1^, the production decreased to moderate levels and fell to low levels at 1.5 mol L^−1^ NaCl. However, PS104 was sensitive to salt stress, with its HCN production being inhibited at 0.25 mol L^−1^ NaCl. These findings highlight the variability in bacterial strains’ responses to salinity, emphasizing the exceptional tolerance of some strains, such as PS36, compared to the increased sensitivity of others, including PS104 and PS154.

All the tested strains PS36, PS104, and PS154 were NH_3_ producers across the whole tested salinity gradient. Quantitatively, stability in NH_3_ production has been recorded; all the strains produce this metabolite within the range of 0–1.5 mol L^−1^. i.e., including the highest values of salinity, which could presumably be stressful for bacterial metabolism

Strains PS36 and PS154 maintained siderophore production at all NaCl concentrations tested (0–1.5 mol L^−1^). However, PS104 showed no siderophore production at any NaCl concentration tested, indicating a lack of this property under the salt conditions studied.

For the activity of the enzyme 1-aminocyclopropane-1-carboxylate (ACC) deaminase, the results showed a complete lack of activity for all strains (PS36, PS104, and PS154). This lack of enzymatic activity indicates that ACC deaminase did not degrade ACC (a precursor of ethylene) to detectable levels, suggesting that these strains are unlikely to contribute to ethylene reduction under the tested conditions.

### 3.4. 16S rRNA Barcoding and Phylogenetic Study

The isolates are classified into three distinct genera: *Pseudomonas*, *Bacillus*, and *Niallia*. The phylogenetic trees of the strains PS36, PS104, and PS154 are displayed in Figures S1–S3, respectively. Strain PS36 formed a monophyletic clade with three sequences identified as *Pseudomonas putida* (accessions MN133999.1, JQ782512.1, and KT361502.1). This clade received a high bootstrap value of 99.5%, indicating strong statistical support for the relationship and a high degree of sequence similarity between PS36 and the reference *P. putida* strains. Strain PS104 was placed in a well-supported clade with two reference strains of *Bacillus proteolyticus* (accession numbers PV652493.1 and OK090545.1). This grouping received a bootstrap value of 1.000, indicating maximum support and suggesting a near-identical 16S rRNA gene sequence among these three strains. Strain PS154 clustered with three reference sequences identified as *Niallia nealsonii* (accessions MK874932.1, KC329823.1, and MN540832.1). This grouping was supported by a bootstrap value of 87.8%, indicating a strong phylogenetic relationship and near-identical 16S rRNA gene sequences among the members of the clade. The genetic distance between PS154 and the *N. nealsonii* sequences was low with a bootstrap value of 87.8% supporting for their close evolutionary proximity.

### 3.5. Controlled Condition Experiment on Medicago sativa

The selection of the best formulation was performed using alfalfa as the model plant. A two-way ANOVA was conducted to assess the effects of inoculation, salinity, and their interaction on germination, root length, shoot length, chlorophyll content, and proline accumulation in *Medicago sativa*. Table 4 shows the results obtained for the main effect of Inoculation.

Consortium 1 consistently showed the highest values across all measured parameters. It achieved the most excellent germination rate (91.1%), root length (3.87 cm), shoot length (4.37 cm), and chlorophyll content (13.32 mg/100 g FW) (*p* < 0.05). Proline content was lower in Consortium 1 (2.32 g/g FW) compared to the non-inoculated control (2.98 g/g FW) (*p* < 0.05). As presented in Table, the plant response was different based on salinity concentration.

As shown in Table 5, the highest chlorophyll content was recorded at 50 mM NaCl (14.37 mg/100 g FW) (*p* < 0.05), while shoot length and root length were highest at 100 mM NaCl (4.46 cm and 3.96 cm, respectively) (*p* < 0.05). Germination and chlorophyll content declined progressively at higher salinity levels (200–300 mM NaCl). Proline content increased with salinity, reaching maximum values at 100–200 mM NaCl (2.70–2.72 g/g FW) (*p* < 0.05). The Interaction effect Inoculation × Salinity, reported in Table S4, was also significant. Consortium 1 combined with 50 mM NaCl yielded the highest chlorophyll concentration (15.33 mg/100 g FW). In comparison, the same consortium under 100 mM NaCl exhibited the highest shoot length (4.83 cm), root length (4.33 cm), and elevated germination (93.3%) (*p* < 0.05). Proline levels remained moderate across salinity levels when inoculated with Consortium 1 (2.09–2.61 g/g FW) (*p* < 0.05), in contrast to higher proline values observed in uninoculated plants under similar conditions (up to 3.54 g/g FW at 200 mM NaCl) (*p* < 0.05). Based on these results, the formulation Consortium 1 was further investigated on durum wheat.

### 3.6. Greenhouse Experiment on Triticum durum

The Consortium 1 was tested under greenhouse conditions to evaluate the impact of a bacterial application on the growth of *Triticum durum.* Different salinity levels (0–1500 mM NaCl) were applied. Table 6 reports the results obtained from the trial.

Under non-inoculated conditions, plant growth was maintained up to 500 mM NaCl. The most marked increase in root length due to bacterial inoculation was observed at 0 mM NaCl, where values rose by 111% compared to the control. At 250 mM NaCl, root length increased by 91%. A positive effect was still present at 500 mM, with an increase of 39%. No comparison was possible at 750 mM due to missing values for non-inoculated plants. Shoot length followed a similar trend. The highest improvement was recorded at 500 mM NaCl, with a 112% increase. At 0 mM and 250 mM, shoot length rose by 71% and 59%, respectively. These results confirm that bacterial inoculation promoted shoot development under both optimal and saline conditions. Chlorophyll content increased strongly with bacterial application. At 0 mM and 250 mM NaCl, total chlorophylls rose by 223% and 208%, respectively. At 500 mM, a slight decrease (−15.8%) was recorded in inoculated plants compared to uninoculated ones. The chlorophyll a/b ratio was also affected. At 500 mM NaCl, inoculated plants showed an increase of 116.7% over the uninoculated group. At 0 mM, the ratio changed only slightly (+3.4%), while a sharp reduction (−56.7%) was recorded at 250 mM. Antioxidant activity values (DPPH) decreased in all inoculated treatments, indicating stronger radical scavenging capacity. The most pronounced reduction was observed at 250 mM (−21.9%), followed by 0 mM (−18.4%) and 500 mM (−14.0%). Proline content increased in all inoculated conditions. At 0 mM, levels rose by 45.7%. Increases of 19.9% and 21.9% were recorded at 250 mM and 500 mM, respectively. No plant emergence occurred in treatments E (1500 mM, no bacteria) and J (1500 mM, with bacteria), confirming that 1500 mM NaCl is lethal for this cultivar, regardless of inoculation.

**Figure 5:**
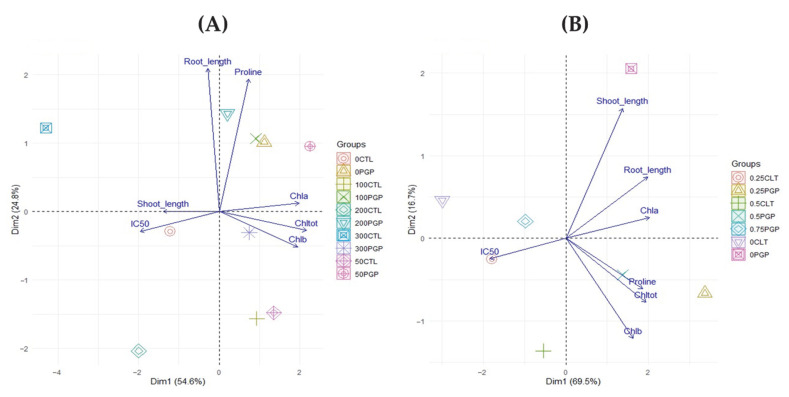
Biplot obtained from the principal component analysis (PCA) for Alfalfa (**A**) and Wheat (**B**).

To highlight possible correlations for in planta outcomes, the results were investigated with principal component analysis (PCA). The analysis revealed a clear separation between inoculated and non-inoculated plants under different salinity levels in both *Medicago sativa* and *Triticum*. In alfalfa (Figure 5A), the first principal component (Dim1, 54.6%) was strongly associated with photosynthetic pigments (Chl a, Chl b, Chl tot) in opposition to IC_50_, while the second component (Dim2, 24.8%) distinguished samples mainly according to root length and proline accumulation. In this system, PGP treatments clustered in the space of chlorophylls and root growth, indicating that bacterial inoculation preserved photosynthetic efficiency and promoted osmotic adjustment, as reflected by proline increase. Control plants, in contrast, were positioned toward IC_50_, reflecting higher cellular stress. In wheat (Figure 5B), group separation was even more pronounced. The first component (Dim1, 69.5%) clearly discriminated against inoculated plants, associated with higher levels of chlorophylls and proline, from controls characterized by higher IC_50_ values. The second component (Dim2, 16.7%) further distinguished samples based on growth parameters, confirming that inoculation supported both root and shoot development. Comparison between the two species highlights different adaptive responses to salinity. In *Medicago*, inoculation primarily enhanced osmotic tolerance and root performance, whereas in *Triticum* the most evident effect was the preservation of photosynthetic functionality. In both cases, the multivariate distribution consistently confirmed the positive role of bacterial inoculation in mitigating the detrimental effects of salinity.

## 4. Discussion

Soil salinity is one of the most widespread environmental stresses impacting agricultural productivity, particularly in semi-arid and arid regions such as the Mediterranean and North Africa. It affects more than 25% of irrigated land globally, causing significant reductions in yield, especially in salt-sensitive crops like *Triticum durum* [6,7]. In this context, the use of plant growth-promoting rhizobacteria (PGPR) offers a promising alternative to chemical soil amendments, contributing to sustainable agriculture while preserving soil biodiversity [12,13]. This study investigated the isolation and selection of halotolerant bacterial strains from an alkaline–saline agricultural soil in Algeria.

The analysis of the 16S rRNA gene amplicons from the Algerian saline-alkaline soil revealed clear shifts in microbial community structure driven by salinity Table 2, Figure 1 and Figure 2. Similarly to patterns observed in soils from the Qaidam Basin and the Yellow River Delta, we found that increased salt levels reduced overall bacterial diversity (alpha and beta diversity) and altered community composition [33,34]. In particular, phyla such as Actinobacteria, Chloroflexi and Acidobacteria were notably less abundant under higher salinity, while Proteobacteria and Bacteroidetes often became more prevalent [35]. Since this study did not include a low-salinity control soil, these observations are interpreted in the context of existing bibliographic data. Notably, the rhizosphere of salt-tolerant plants often harbours halotolerant PGPR, and phyla like Chloroflexi which were present in our samples are frequently associated with nutrient cycling and stress resilience [36]. Our findings support the selection of *Pseudomonas*, *Bacillus* and *Niallia*, genera that are well-known for plant growth promotion, as likely to integrate and persist within the native community. Moreover, this combination of data reinforces the need for a top–down approach. Understanding the baseline microbiome composition via culture-independent methods (such as 16S sequencing) informs targeted culture-dependent isolation strategies, ensuring that selected strains are ecologically compatible and effective in their natural context [37]. Interestingly, the most effective PGPR strains selected in this study did not belong to the dominant phyla revealed by 16S rRNA metabarcoding analysis. This discrepancy can be explained by the specific isolation conditions employed, including selective media composition, salt concentration, and incubation parameters, which may favour the growth of halotolerant strains with plant growth-promoting traits over more abundant but less stress-tolerant community members. Recent high-throughput sequencing studies of saline soils reveal that these environments host microbial communities dominated by Proteobacteria, Actinobacteria, and Bacteroidota, with genera such as *Pseudomonas* being prevalent in the rhizosphere. Additionally, cultivable isolates include halotolerant Bacilli/*Peribacillus* strains that carry osmoprotective genes (otsA/otsB). These findings, consistent with the observed growth enhancement under saline conditions after inoculation, highlight that saline soils are rich reservoirs of beneficial microorganisms with significant potential for biofertilization [38].

The selected strains *Pseudomonas putida* (PS36), *Bacillus proteolyticus* (PS104), and *Niallia nealsonii* (PS154) exhibited robust growth-promoting traits under a range of salt concentrations Table 3, Figure 3, making them suitable candidates for the formulation of a bacterial consortium aimed at improving wheat growth under salinity stress. The three strains maintained their capacity to produce phosphate-solubilising enzymes, gibberellic acid, indoles (IAA equivalents), and ammonia across a salinity gradient from 0 to 1.5 mol L^−1^ NaCl. In particular, *N. nealsonii* PS154 demonstrated stable and even enhanced indole production under higher salt conditions Figure 4, suggesting a stress-induced upregulation of auxin biosynthesis, which may play a central role in root development and stress mitigation [39,40].This observation aligns with previous findings by [41,42] who reported increased auxin production in halotolerant PGPR at moderate to high salinity levels. PS36 maintained strong hydrogen cyanide (HCN) production under salt stress up to 0.75 mol L^−1^, while PS104 and PS154 were more sensitive, losing HCN synthesis capacity at relatively low salt concentrations. This variability highlights the strain-specific responses to environmental conditions, as noted by [43,44]. Although HCN can be phytotoxic at high concentrations, in low amounts it may enhance nutrient uptake and stimulate ethylene-mediated stress responses [45,46]. Ammonia production was sustained in all three strains across all salt concentrations Table 3. This trait is particularly valuable in saline soils where nitrogen availability is limited due to osmotic imbalances and reduced microbial activity. PGPR-mediated ammonia production has been linked to improved nitrogen cycling and enhanced resistance to both abiotic and biotic stresses [47,48]. In addition, both PS36 and PS154 exhibited stable siderophore production under salinity Table 3, facilitating iron acquisition in calcareous, alkaline soils. Siderophores not only improve plant micronutrient uptake but also help modulate oxidative stress responses under salinity [49,50].

Numerous studies have highlighted the role of *Pseudomonas*, *Bacillus*, and, more recently, *Niallia* as plant growth-promoting bacteria, particularly under saline stress conditions where conventional crop performance is severely compromised. *Pseudomonas* species are well-documented for their remarkable metabolic versatility and the production of key compounds such as siderophores, ACC deaminase, indole-3-acetic acid (IAA), and various antimicrobial metabolites that inhibit phytopathogens and stimulate root development [51,52]. Their capacity to accumulate compatible osmolytes, such as proline and glycerol, along with their ability to modify membrane lipid composition under high salinity, contributes to osmotic adjustment and cellular homeostasis [53]. In parallel, *Bacillus* spp. demonstrate strong ecological resilience, supported by their capacity for endospore formation, the production of antioxidant enzymes (e.g., peroxidases, superoxide dismutase), and the secretion of extracellular polymers that improve soil structure and plant tolerance to salt stress [54,55]. Additionally, the Na^+^/H^+^ antiporters encoded by *Bacillus* play a critical role in maintaining ionic balance by removing excess sodium from the cytoplasm, thus preventing its toxic accumulation. *Niallia*, a recently reclassified genus formerly grouped under *Bacillus*, is emerging as a promising PGPR. Preliminary studies suggest its ability to produce auxins, hydrolytic enzymes, and volatile organic compounds (VOCs) involved in plant growth promotion and systemic defense [56,57]. However, the current literature on *Niallia* remains limited, emphasizing the need for further comparative investigations to fully assess its functional capacity relative to other PGPR genera. In this context, understanding the hormonal responses of plants, particularly the role of ethylene, is essential to complement the physiological insights provided by PGPR action under saline conditions. Ethylene is a key phytohormone involved in plant adaptation to both water and salt stress, notably by regulating stomatal closure, modulating root architecture, and promoting mechanisms that minimize water loss and optimize nutrient uptake [58]. Under saline conditions, ethylene also plays a protective role by regulating growth and limiting the accumulation of toxic ions through exclusion processes [59]. However, its effects are concentration-dependent and can become inhibitory under excessive accumulation. Notably, several PGPR strains produce 1-aminocyclopropane-1-carboxylate (ACC) deaminase, an enzyme that lowers plant ethylene levels by degrading its precursor, ACC. This regulation may improve plant tolerance to abiotic stress, yet its efficacy can vary depending on environmental context and host–plant interactions. None of the studied PGP strains produced ACC deaminase, a trait often linked to stress mitigation via ethylene regulation. This may reflect the selective pressure of the saline-alkaline soil, where other traits such as halotolerance, osmoprotectant synthesis, or antioxidant production could be more relevant. The strains may also regulate plant ethylene levels through alternative mechanisms, highlighting the importance of considering multiple functional traits when selecting PGPR for saline soils. [60,61]. These observations suggest that the ethylene-mediated response to salinity is more complex than previously assumed, underlining the need for deeper investigations into hormonal signaling pathways under combined water and salt stress. Taken together, these three genera are increasingly being integrated into synthetic or native microbial consortia, with several recent studies and meta-analyses reporting synergistic effects on nutrient cycling, plant stress resilience, and biomass enhancement [62,63,64].

The in planta trials carried out on Alfalfa and wheat underline the potential of these strains (particularly Consortium 1) as a viable solution for promoting crop growth and resilience in salt-affected soils, supporting their broader application in sustainable agriculture. This aspect was also highlighted by PCA, which showed the correlations between the treatments in the presence of salt stress and the investigated variables. The distinct clustering of inoculated plants suggests that the bacterial consortium positively influences multiple physiological traits in an integrated manner, which contributes to improved stress tolerance. The separation between inoculated and uninoculated plants highlights the effectiveness of the microbial treatment in modulating plant responses to salinity. As reported in the literature, the PCA confirms that the synergistic interactions within the bacterial consortium may optimize photosynthetic efficiency, osmotic regulation, and oxidative stress management, which are crucial factors for plant survival in saline environments [62,63,64]. In controlled experiments on *M. sativa*, the bacterial consortium significantly enhanced germination, root and shoot length, and chlorophyll content under varying salt conditions. The observed increase in proline content in inoculated plants, although lower than in uninoculated controls, suggests a more efficient osmoregulatory mechanism that reduces the need for excessive proline accumulation a known stress marker [30,65]. Plants employ amino acids such as proline to maintain cellular osmotic and antioxidant balance during abiotic stress. In addition to being an osmoprotectant, proline can operate as a stress signal; hence, plants may acquire more proline due to abiotic stress or less proline as a result of decreased stress [14]. These promising results were further validated in a greenhouse experiment using *T. durum* as the target crop Table 6. In the presence of the bacterial inoculum, plants exhibited significantly improved shoot and root growth, even at 750 mM NaCl. Chlorophyll content, a proxy for photosynthetic efficiency, was highest in inoculated plants under 250–500 mM salinity. Despite the disruption of the Chla/Chl b ratio registered for the different salt concentration, PGPR inoculation clearly enhanced photosynthetic performance. These physiological improvements likely reflect better osmotic adjustment, nutrient uptake, and stress hormone modulation [66,67]. The increase in antioxidant activity in uninoculated plants under salt stress, contrasted with lower IC50 values in inoculated groups, supports the hypothesis that PGPR inoculation reduces oxidative damage, allowing for more efficient energy allocation to growth [32]. Similarly, elevated proline levels in inoculated plants confirm that the consortium supports osmoregulation and cellular protection mechanisms under salinity, as previously reported by [68,69].

Despite the benefits, the study identified a biological threshold beyond which the inoculation was no longer effective. At 1500 mM NaCl, no plant growth was observed, regardless of bacterial presence. This indicates that the physiological limits of *T. durum* are surpassed at this salinity, and microbial support alone cannot compensate for the osmotic and ionic stresses incurred. Similar constraints have been reported in other salt-sensitive crops [55,70]. Another limitation concerns the scalability and transferability of these results to field conditions. Although the greenhouse setup provides valuable insights, the performance of bioinoculants is influenced by multiple environmental variables, including soil texture, native microbial communities, and irrigation practices [71]. Moreover, the interactions between the introduced strains and the indigenous microbiota were not assessed, which could affect colonisation and persistence in real-world applications.

The formulation of a multi-strain bacterial consortium presents a viable strategy for mitigating salinity stress in wheat cultivation, particularly in marginal soils typical of North African agricultural zones. The strains used are native to the target environment, which may favour their adaptation and long-term persistence. However, successful implementation at scale will require further investigation. This includes: (i) field trials under varying climatic and soil conditions; (ii) evaluation of formulation carriers and storage stability; assessment of long-term effects on soil health and crop productivity; (iii) economic analysis of production and application costs. While chemical amendments such as gypsum and lime remain common, they are often unsustainable and environmentally taxing. In contrast, bio-based solutions like the consortium described in this study align with the principles of regenerative agriculture and offer a pathway to improving resilience in food systems under climate stress [72,73].

## 5. Conclusions

This study highlights the potential of a native bacterial consortium comprising *Pseudomonas putida*, *Bacillus proteolyticus*, and *Niallia nealsonii* to mitigate the adverse effects of salinity on wheat growth. The selected strains demonstrated key plant growth-promoting activities under high salt conditions and, when applied as a consortium, significantly improved physiological and morphological parameters in both *Medicago sativa* and *Triticum durum*. The inoculated plants maintained chlorophyll levels, accumulated proline efficiently, and exhibited better root and shoot development under salt stress up to 750 mM NaCl. These findings confirm the functional resilience of the microbial consortium and its relevance in enhancing crop performance in saline soils. However, the benefits declined at extreme salinity levels, indicating the importance of combining microbial strategies with other agronomic measures in severely affected environments. While greenhouse results are promising, further field-based studies are needed to validate the practical application of this bioformulation, particularly under variable climatic and edaphic conditions. Future studies should focus on field experiments conducted in zones with varying levels of salinity. Moreover, investigating its potential synergy with other agronomic practices could enhance its utility for dealing with severe salinity. Longer experiments investigating its effect on soil quality and microbial communities under prolonged salt stress would also be necessary to evaluate its sustainability and potential future application. The use of native halotolerant bacteria represents a viable, sustainable alternative to conventional soil amendments and aligns with broader goals of improving food security and soil health in salt-impacted agricultural regions.

## Figures and Tables

**Table 1:** The various treatments compared for alfalfa.

Treatments		
A. no bacteria and no salt stress		
B. no bacteria and salt stress 50 mM		
C. no bacteria and salt stress 100 mM		
D. no bacteria and salt stress 200 mM		
E. no bacteria and salt stress 300 mM		
Consortium 1	Consortium 2	Consortium 3
F1. 10^6^ CFU mL^−1^ bacteria and no salt stress	F2. 10^6^ CFU mL^−1^ bacteria and no salt stress	F3. 10^6^ CFU mL^−1^ bacteria and no salt stress
G1. 10^6^ CFU mL^−1^ bacteria and salt stress 50 mM	G2. 10^6^ CFU mL^−1^ bacteria and salt stress 50 mM	G3. 10^6^ CFU mL^−1^ bacteria and salt stress 50 mM
H1. 10^6^ CFU mL^−1^ bacteria and salt stress 100 mM	H2. 10^6^ CFU mL^−1^ bacteria and salt stress 100 mM	H3. 10^6^ CFU mL^−1^ bacteria and salt stress 100 mM
I1. 10^6^ CFU mL^−1^ bacteria and salt stress 200 mM	I2. 10^6^ CFU mL^−1^ bacteria and salt stress 200 mM	I3. 10^6^ CFU mL^−1^ bacteria and salt stress 200 mM
J1. 10^6^ CFU mL^−1^ bacteria and salt stress 300 mM	J2. 10^6^ CFU mL^−1^ bacteria and salt stress 300 mM	J3. 10^6^ CFU mL^−1^ bacteria and salt stress 300 mM

**Table 2:** Bacterial community profile characterized through 16S rRNA gene metabarcoding.

Index	Taxa	Reads	Dominance	Simpson	Shannon	Evenness	Chao-1
Value	77	14,948	0.01985	0.9801	4.097	0.7815	77

**Table 3:** Summary of the HCN, NH_3_, and siderophores results obtained for PS36, PS104, and PS154 strains. For each parameter: +++, high production; ++, medium production; +, low production; −, no production.

NaCl Concentration	Test	PS36	PS104	PS154
0 moL/L NaCl	HCN	+++	++	+++
	NH_3_	+	+	+
	Siderophores	+	−	+
0.25 moL/L NaCl	HCN	+++	−	−
	NH_3_	+	+	+
	Siderophores	+	−	+
0.5 moL/L NaCl	HCN	+++	−	−
	NH_3_	+	+	+
	Siderophores	+	−	+
0.75 moL/L NaCl	HCN	+++	−	−
	NH_3_	+	+	+
	Siderophores	+	−	+
1 moL/L NaCl	HCN	++	−	−
	NH_3_	+	+	+
	Siderophores	+	−	+
1.25 moL/L NaCl	HCN	++	−	−
	NH_3_	+	+	+
	Siderophores	+	−	+
1.5 moL/L NaCl	HCN	+	−	−
	NH_3_	+	+	+
	Siderophores	+	−	+

**Table 4:** Differences in plant growth and stress parameters of *M. sativa* among treatments based on the Inoculation factor with a 95% confidence interval. Results followed by the same letter are not significantly different according to Fisher’s Least Significant Difference (LSD) post hoc test n = 3.

Treatment	Germination (%)	Root Length (cm)	Shoot Length (cm)	Chlorophylls (mg/100 g FW)	Proline (g/g FW)
Consortium 1	91.1 ± 1.21 a	3.87 ± 0.27 a	4.37 ± 0.30 a	13.32 ± 0.44 a	2.32 ± 0.48 b
Consortium 2	83.3 ± 0.15 b	3.47 ± 0.23 b	3.97 ± 0.29 b	12.01 ± 0.54 b	2.17 ± 0.38 c
Consortium 3	80.7 ± 0.10 c	3.34 ± 0.23 b	3.84 ± 0.29 b	11.59 ± 0.24 c	2.11 ± 0.32 c
No	83.6 ± 0.06 b	3.16 ± 0.19 b	3.66 ± 0.15 b	10.72 ± 0.24 d	2.98 ± 0.51 a
LSD-value	1.01	0.37	0.36	0.32	0.12

**Table 5:** Differences in plant growth and stress parameters of *M. sativa* among treatments based on the Salinity factor with a 95% confidence interval. Results followed by the same letter are not significantly different according to Fisher’s Least Significant Difference (LSD) post hoc test n = 3.

Treatment	Germination (%)	Root Length (cm)	Shoot Length (cm)	Chlorophylls (mg/100 g FW)	Proline (g/g FW)
0	91.8 ± 0.58 a	3.20 ± 0.44 b	3.70 ± 0.17 b	12.19 ± 0.74 b	1.60 ± 0.45 c
50	89.8 ± 0.58 b	3.40 ± 0.29 b	3.90 ± 0.17 b	14.37 ± 0.56 a	2.33 ± 0.67 b
100	87.3 ± 0.58 c	3.96 ± 0.17 b	4.46 ± 0.44 a	12.16 ± 0.92 b	2.70 ± 0.47 a
200	79.8 ± 0.58 d	3.57 ± 0.33 ab	4.07 ± 0.00 ab	10.98 ± 0.24 c	2.72 ± 0.41 a
300	74.8 ± 0.58 e	3.18 ± 0.58 b	3.68 ± 0.33 b	11.10 ± 0.24 c	2.62 ± 0.93 a
LSD-value	1.1	0.42	0.43	0.35	0.14

**Table 6:** Differences in plant growth and stress parameters of *T. durum* among treatments based on factors and their interaction with a 95% confidence interval. Results followed by the same letter are not significantly different according to Fisher’s Least Significant Difference (LSD) post hoc test.

Parameter	A	B	C	F	G	H	I				
Root Lenght	3.10 d	3.47 cd	3.77 c	6.53 a	6.63 a	5.23 b	5.00 b	Significance	no	***	***
								LSD-value	-	0.48	0.63
Shoot Lenght	4.67 cd	3.83 d	2.50 e	8.00 a	6.10 b	5.30 bc	3.83 d	Significance	no	***	***
								LSD-value	-	0.80	0.63
Chlorophylls	10.39 e	20.05 d	43.78 b	33.57 c	61.80 a	36.86 c	20.34 d	Significance	no	***	***
								LSD-value	-	2.73	3.62
Chla/Chlb	2.28 a	2.03 a	0.92 b	2.36 a	0.88 b	1.99 a	1.32 b	Significance	no	***	***
								LSD-value	-	0.47	0.63
Antioxidant activity	25.37 d	36.71 a	26.84 c	20.71 f	28.67 b	23.09 e	21.65 f	Significance	no	***	***
								LSD-value	-	0.87	1.15
Proline	7.47 e	9.63 d	10.24 cd	10.89 bc	11.55 b	12.48 a	9.76 d	Significance	no	***	***
								LSD-value	-	0.69	1.15

In the table: **A**: No bacteria and no salt stress, **B**: No bacteria and salt stress 250 mM, **C**: No bacteria and salt stress 500 mM, **F**: Bacteria and no salt stress, **G**: Bacteria and salt stress 250 mM, **H**: Bacteria and salt stress 500 mM, **I**: Bacteria and salt stress 750 mM (see also Section 2.7). *** *p* ≤ 0.001.

## Data Availability

The original contributions presented in this study are included in the article/Supplementary Material. Further inquiries can be directed to the corresponding author.

## Supplementary Materials

The following supporting information can be downloaded at: https://www.mdpi.com/article/10.3390/microorganisms13102273/s1. Table S1: Order assignments of unknown families; Table S2: Family assignments of unknown genera; Table S3: Ranking analysis results; Table S4: Differences in plant growth and stress parameters of *M. sativa* among treatments in the Inoculation Salinity interaction with a 95% confidence interval; Table S5: Changes in plant growth and stress parameters from uninoculated to inoculated plants at the same salinity level of *T. durum*. Figures S1–S3: Phylogenetic trees inferred for strains PS36, PS104, and PS154, respectively, using the Maximum Likelihood method.
