# Supplementary material for: Selection of a Bacterial Conditioner to Improve Wheat Production Under Salinity Stress
Source: Microorganisms. 2025 Sep 28;13(10):2273. doi: 10.3390/microorganisms13102273 (PMC12565890; doi:10.3390/microorganisms13102273)
Supplement: Supplementary file 1 [file microorganisms-13-02273-s001.zip › microorganisms-3858175-supplementary.pdf]

Table S1. Order assignments of unknown families

Unknown Families				
**Kingdom**	**Phylum**	**Class**	**Order**	**Relative abundance (%)**
Bacteria	Chloroflexi	Ktedonobacteria	C0119	4.1
Bacteria	Cyanobacteria	Cyanobacteriia	Chloroplast	0.2
Bacteria	Verrucomicrobiota	Verrucomicrobiae	Chthoniobacterales	0.4
Bacteria	Armatimonadota	Chthonomonadetes	Chthonomonadales	0.4
Bacteria	Proteobacteria	Alphaproteobacteria	Elsterales	1.1
Bacteria	Actinobacteriota	Thermoleophilia	Gaiellales	4.2
Bacteria	Proteobacteria	Alphaproteobacteria	Unknown	17.7
Bacteria	Chloroflexi	Anaerolineae	RBG-13-54-9	0.3
Bacteria	Acidobacteriota	Vicinamibacteria	Vicinamibacterales	0.9

Table S2. Family assignments of unknown genera

Unknown Genera					
**Kingdom**	**Phylum**	**Class**	**Order**	**Family**	**Relative abundance (%)**
Bacteria	Proteobacteria	Alphaproteobacteria	Acetobacterales	Acetobacteraceae	2.4
Bacteria	Acidobacteriota	Acidobacteriae	Acidobacteriales	Acidobacteriaceae (Subgroup 1)	0.5
Bacteria	Firmicutes	Bacilli	Bacillales	Bacillaceae	1.9
Bacteria	Proteobacteria	Alphaproteobacteria	Rhizobiales	Beijerinckiaceae	3.1
Bacteria	Chloroflexi	Anaerolineae	Caldilineales	Caldilineaceae	0.4
Bacteria	Bacteroidota	Bacteroidia	Chitinophagales	Chitinophagaceae	1.7
Bacteria	Entotheonellaeota	Entotheonellia	Entotheonellales	Entotheonellaceae	0.5
Bacteria	Chloroflexi	Ktedonobacteria	Ktedonobacterales	JG30-KF-AS9	2.6
Bacteria	Chloroflexi	Chloroflexia	Thermomicrobiales	JG30-KF-CM45	0.3
Bacteria	Chloroflexi	Ktedonobacteria	Ktedonobacterales	Ktedonobacteraceae	0.4
Bacteria	Bacteroidota	Bacteroidia	Cytophagales	Microscillaceae	0.9
Bacteria	Proteobacteria	Alphaproteobacteria	NA	NA	14.5
Bacteria	Verrucomicrobiota	Verrucomicrobiae	Pedosphaerales	Pedosphaeraceae	2.8
Bacteria	Actinobacteriota	Actinobacteria	Streptomycetales	Streptomycetaceae	1.8
Bacteria	Proteobacteria	Alphaproteobacteria	Rhizobiales	Xanthobacteraceae	6.1
Bacteria	Patescibacteria	Saccharimonadia	Saccharimonadales	YM_S32_TM7_50_20	0.4

Table S3. Ranking analysis results.

Strain	Phosphate solubilization	Giberellic Acid	Indoles	Rank Phosphate	Rank Giberellic Acid	Rank Indoles	Total Score
**PS104**	93.11	56.41	66.57	6	2	3	11

**PS154**	147.49	31.52	71.41	3	21	2	26
**PS36**	194.13	21.51	72.81	1	32	1	34
**PS41**	76.46	31.62	66.57	13	19	3	35
**PS105**	183.58	46.76	28.61	2	3	34	39
**PS190**	60.28	56.59	28.99	24	1	31	56
**PS97**	84.17	31.62	28.84	9	19	32	60
**PS25**	48.23	32.10	35.01	37	18	18	73
**PS99**	40.67	33.22	51.90	57	16	6	79
**PS169′**	119.90	18.38	28.58	4	42	36	82
**PS187**	65.34	43.17	10.90	18	5	78	101
**PS96**	55.95	18.91	27.42	27	39	39	105
**PS98**	33.09	26.42	64.45	77	26	5	108
**PS94**	50.96	11.29	50.07	35	68	8	111
**PS133**	35.61	34.88	29.40	72	14	28	114
**PS188**	40.62	34.93	25.80	59	13	42	114
**PS86**	46.75	11.55	45.92	42	66	10	118
**PS147**	52.61	12.58	31.86	33	61	24	118
**PS177**	22.75	44.93	36.06	99	4	16	119
**PS208**	40.67	27.61	26.97	58	24	40	122
**PS153**	38.82	19.71	33.07	63	37	22	122
**PS182**	44.54	22.69	23.95	47	30	46	123
**PS87**	47.04	11.52	35.01	40	67	18	125
**PS209**	56.89	16.69	20.70	26	48	55	129
**PS193**	60.28	36.04	4.43	24	9	101	134
**PS164**	15.18	35.98	44.55	111	11	13	135
**PS148**	23.62	26.22	45.51	96	28	11	135
**PS131**	38.46	36.04	14.73	65	9	67	141
**B4**	74.01	7.20	33.45	15	105	21	141
**PS43**	72.23	33.22	3.45	16	16	112	144
**PS181**	80.86	14.21	10.00	11	56	81	148
**PS85**	45.84	12.00	25.48	44	63	43	150
**PS64′**	94.74	7.85	21.37	5	95	51	151
**PS63**	62.84	6.65	31.86	21	108	24	153
**PS225**	27.46	35.83	17.58	85	12	60	157
**PS103**	22.42	33.42	26.78	101	15	41	157
**PS163**	37.99	21.22	17.39	66	33	61	160
**PS224**	19.58	39.58	25.06	107	8	45	160
**PS155**	46.11	21.07	8.89	43	34	84	161
**PS137**	42.54	7.53	50.07	54	101	8	163
**PS146**	33.01	14.06	28.84	78	58	32	168
**PS15**	41.98	15.03	17.13	55	54	62	171
**PS183**	36.54	40.81	4.22	69	6	102	177
**PS207**	23.83	12.77	32.45	95	60	23	178
**PS176**	51.09	8.03	21.02	34	93	52	179
**PS156**	20.77	21.56	23.31	103	31	48	182
**PS132**	48.08	4.96	35.01	38	126	18	182
**PS65′**	40.09	6.49	45.51	62	112	11	185
**PS159**	64.35	11.29	4.10	19	68	104	191
**PS169**	31.38	26.85	7.49	79	25	88	192
**B9**	91.89	7.85	7.24	8	95	90	193
**PS223**	8.52	18.88	28.57	118	40	37	195
**PS62**	43.22	5.80	30.77	52	118	26	196
**PS191**	40.62	9.87	16.92	59	75	63	197
**PS227**	24.88	20.23	13.93	92	35	70	197
**PS143**	44.45	10.25	11.56	49	72	76	197
**PS195**	34.22	40.25	3.18	73	7	117	197
**B8**	45.18	8.91	14.34	46	84	69	199
**PS59**	0.00	11.85	39.87	122	64	15	201
**B3**	29.98	11.85	18.94	81	64	57	202
**PS144**	36.53	13.80	11.63	70	59	74	203
**B2**	54.55	9.40	5.85	30	80	94	204
**B19**	62.28	6.00	14.94	22	117	66	205
**PS49**	47.58	4.04	28.08	39	128	38	205
**PS157**	47.02	18.12	3.04	41	43	122	206
**B5**	70.83	8.73	4.17	17	86	103	206
**PS55**	55.34	9.40	4.87	29	80	97	206

**PS65**	10.49	9.85	36.06	116	76	16	208
**PS95**	0.00	27.64	15.61	122	22	65	209
**PS221**	6.13	19.74	20.80	120	36	54	210
**B10**	74.88	9.85	3.08	14	76	121	211
**PS168**	37.10	17.25	4.78	68	45	99	212
**PS166**	44.05	15.08	3.47	50	53	111	214
**B16**	55.34	11.07	3.24	28	70	116	214
**B15**	53.05	0.55	21.46	32	133	50	215
**PS232**	30.06	27.64	3.31	80	22	113	215
**PS226**	0.00	17.53	22.44	122	44	49	215
**PS165**	92.00	7.63	3.90	7	100	108	215
**PS34**	63.37	9.54	3.10	20	79	119	218
**PS75**	0.00	11.07	29.40	122	70	28	220
**PS180**	49.90	14.21	1.90	36	56	130	222
**PS138**	28.08	18.69	4.87	84	41	97	222
**PS60**	43.34	7.20	14.73	51	105	67	223
**PS26**	38.58	24.71	0.00	64	29	132	225
**PS3**	34.20	8.41	18.22	74	92	59	225
**B18**	54.44	7.40	6.04	31	102	93	226
**PS66′**	28.45	1.59	44.55	83	131	13	227
**PS210**	20.58	16.54	13.62	106	50	72	228
**PS64**	40.92	8.91	7.49	56	84	88	228
**PS189**	20.72	17.11	10.37	104	46	79	229
**PS48**	77.68	6.03	4.10	12	115	104	231
**PS66**	43.22	7.72	8.10	52	97	85	234
**PS179**	26.89	12.57	7.89	87	62	86	235
**B11**	81.27	6.49	3.26	10	112	115	237
**PS77**	24.77	8.56	18.30	93	89	58	240
**PS78**	26.13	7.40	21.02	88	102	52	242
**PS178**	44.49	7.23	4.94	48	104	95	247
**PS161′**	29.51	5.46	25.27	82	122	44	248
**PS158**	35.83	10.11	4.05	71	73	106	250
**PS206**	20.80	19.60	3.65	102	38	110	250
**PS35**	0.00	15.27	11.56	122	52	76	250
**PS42**	19.16	26.42	3.18	108	26	117	251
**PS2**	26.05	7.63	16.92	89	99	63	251
**PS145**	8.04	4.41	51.90	119	127	6	252
**PS192**	20.72	7.89	19.71	104	94	56	254
**PS14**	33.51	16.89	0.86	76	47	131	254
**PS61**	0.00	8.73	23.95	122	86	46	254
**PS161**	40.55	9.58	3.10	61	78	119	258
**PS89**	61.81	6.53	2.19	23	110	128	261
**PS139**	0.00	5.73	30.77	122	121	26	269
**PS1**	12.28	15.88	4.05	115	51	106	272
**PS4**	34.05	5.76	9.07	75	120	83	278
**PS205**	25.76	14.87	0.00	91	55	132	278
**PS27**	0.00	16.64	3.90	122	49	108	279
**PS222**	0.00	2.39	29.33	122	130	30	282
**PS13**	18.67	8.58	7.89	109	88	86	283
**PS233**	0.00	9.07	10.33	122	82	80	284
**PS69**	0.00	0.55	28.61	122	133	34	289
**B12**	37.44	7.72	2.54	67	97	126	290
**PS230**	9.60	8.55	9.57	117	91	82	290
**B13**	45.63	5.16	2.99	45	124	123	292
**PS67**	23.55	5.16	13.78	97	124	71	292
**PS5**	12.90	6.87	11.63	114	107	74	295
**PS47**	0.00	9.07	6.66	122	82	91	295
**PS228**	6.13	6.18	11.74	120	114	73	307
**B17**	24.35	8.56	2.42	94	89	127	310
**B6**	22.56	5.80	4.72	100	118	100	318
**PS0**	25.82	6.03	3.31	90	115	113	318
**PS160**	0.00	10.05	2.71	122	74	125	321
**B7**	22.97	6.65	2.99	98	108	123	329
**B14**	14.96	1.59	6.35	112	131	92	335
**PS53**	27.00	5.46	2.10	86	122	129	337
**PS177′**	12.92	4.04	4.91	113	128	96	337

**PS54**	17.61	6.53	0.00	110	110	132	352

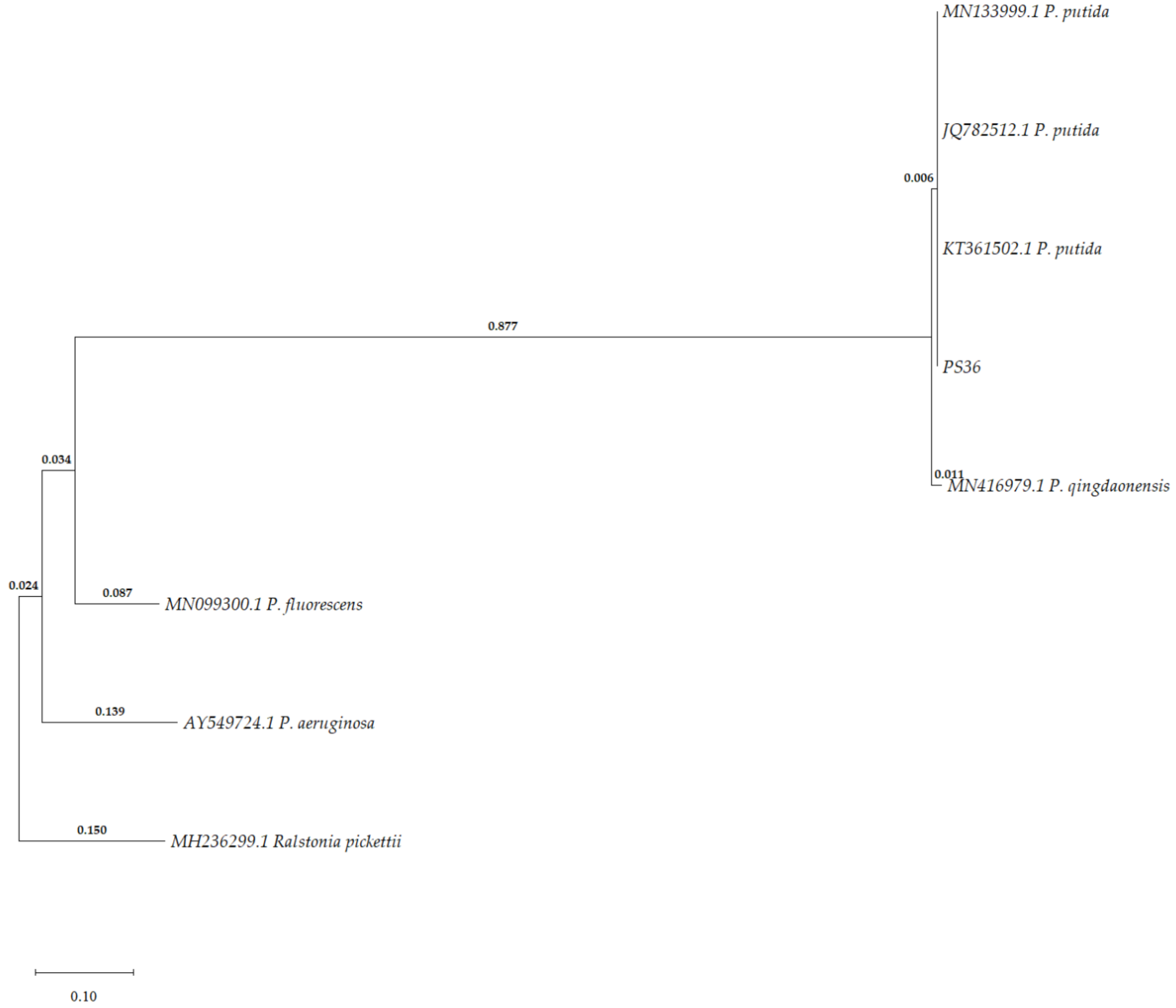

Figure S1. Phylogenetic tree inferred for strain PS36. The evolutionary history was inferred by using the Maximum Likelihood method and Hasegawa-Kishino-Yano model. The tree with the highest log likelihood (-1697.86) is shown. Initial tree(s) for the heuristic search were obtained automatically by applying Neighbor-Join and BioNJ algorithms to a matrix of pairwise distances estimated using the Maximum Composite Likelihood (MCL) approach, and then selecting the topology with superior log likelihood value. The tree is drawn to scale, with branch lengths measured in the number of substitutions per site (above the branches). This analysis involved 8 nucleotide sequences. All positions with less than 95% site coverage were eliminated, i.e., fewer than 5% alignment gaps, missing data, and ambiguous bases were allowed at any position (partial deletion option). There were 419 positions in the final dataset. Evolutionary analyses were conducted in MEGA11.

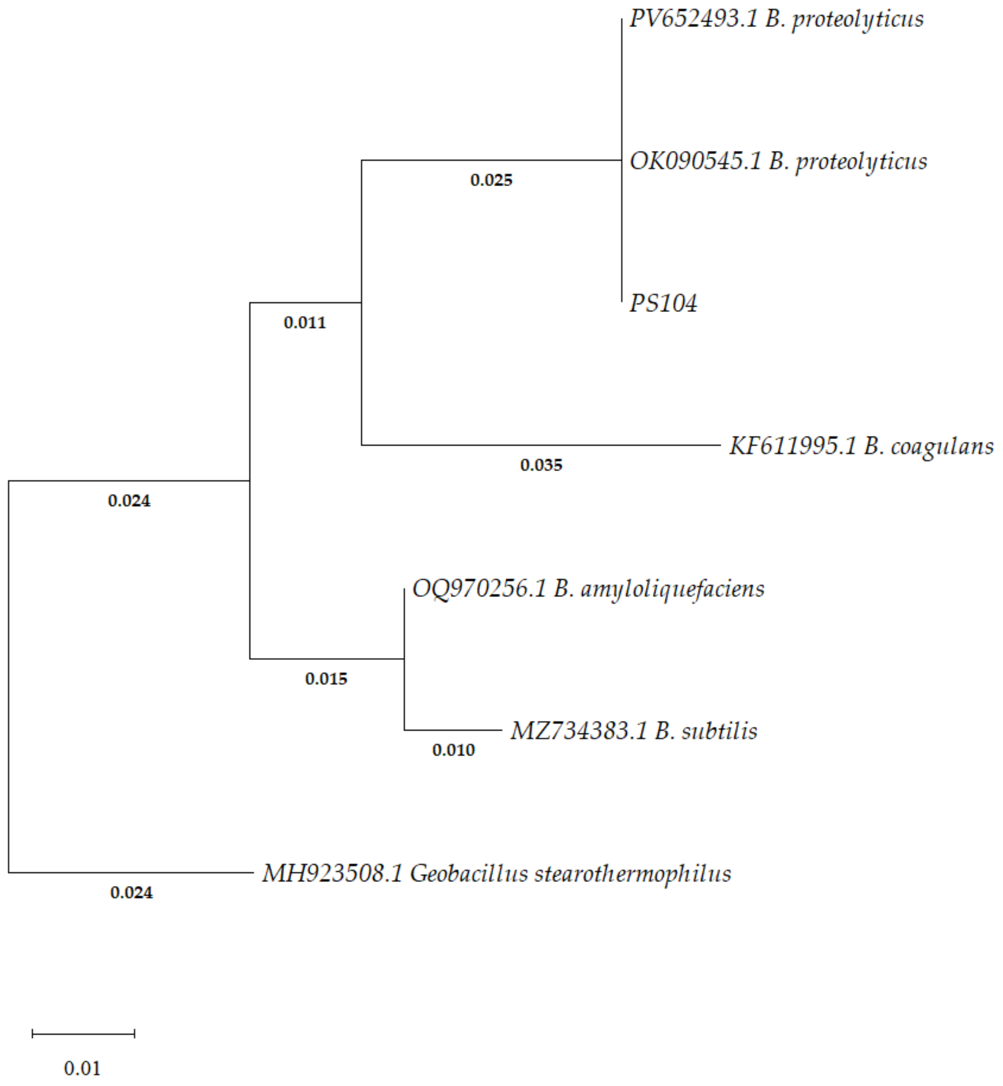

Figure S2. Phylogenetic tree inferred for strain PS104. The evolutionary history was inferred by using the Maximum Likelihood method and Kimura 2-parameter model. The tree with the highest log likelihood (-885.28) is shown. Initial tree(s) for the heuristic search were obtained automatically by applying Neighbor-Join and BioNJ algorithms to a matrix of pairwise distances estimated using the Maximum Composite Likelihood (MCL) approach, and then selecting the topology with superior log likelihood value. The tree is drawn to scale, with branch lengths measured in the number of substitutions per site (above the branches). This analysis involved 7 nucleotide sequences. All positions with less than 95% site coverage were eliminated, i.e., fewer than 5% alignment gaps, missing data, and ambiguous bases were allowed at any position (partial deletion option). There were 417 positions in the final dataset. Evolutionary analyses were conducted in MEGA11.

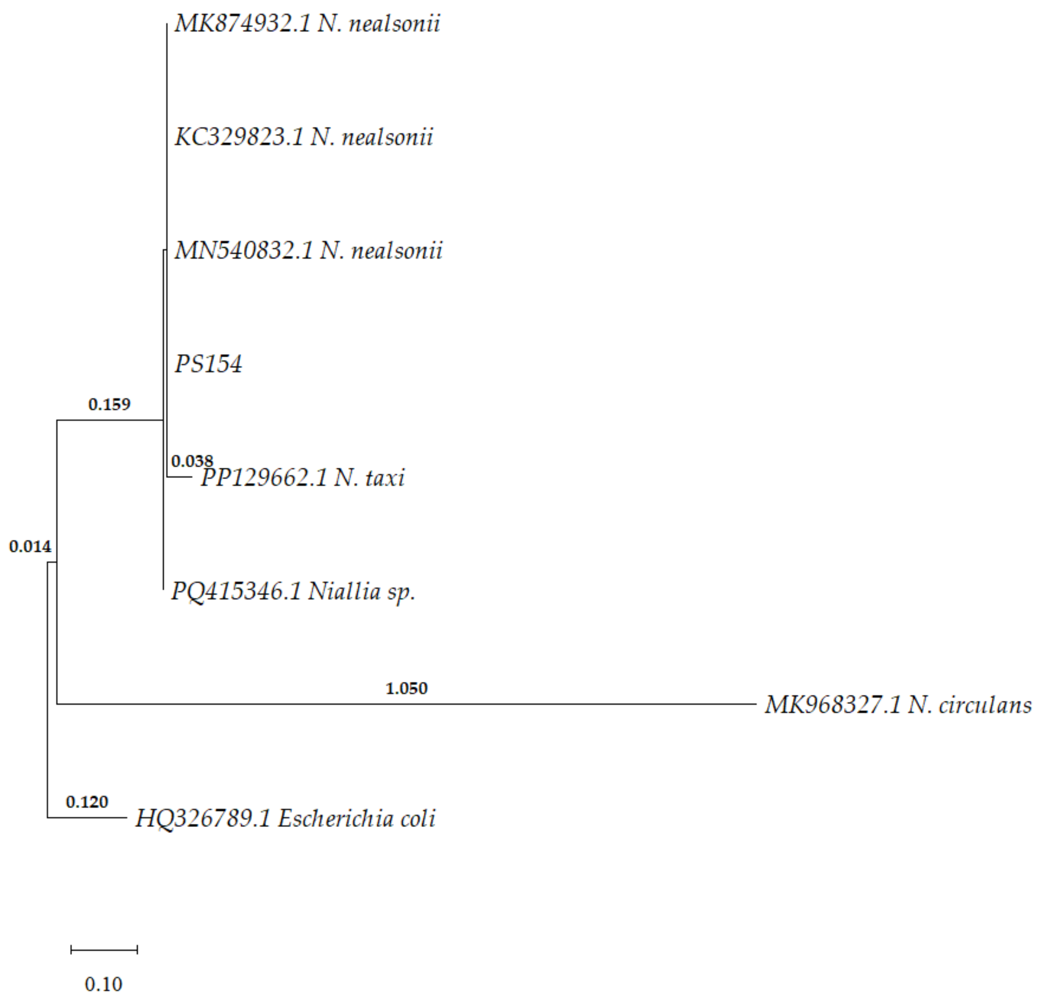

Figure S3. Phylogenetic tree inferred for strain PS154. The evolutionary history was inferred by using the Maximum Likelihood method and Kimura 2-parameter model. The tree with the highest log likelihood (-1557.20) is shown. Initial tree(s) for the heuristic search were obtained automatically by applying Neighbor-Join and BioNJ algorithms to a matrix of pairwise distances estimated using the Maximum Composite Likelihood (MCL) approach, and then selecting the topology with superior log likelihood value. The tree is drawn to scale, with branch lengths measured in the number of substitutions per site (above the branches). This analysis involved 8 nucleotide sequences. All positions with less than 95% site coverage were eliminated, i.e., fewer than 5% alignment gaps, missing data, and ambiguous bases were allowed at any position (partial deletion option). There were 427 positions in the final dataset. Evolutionary analyses were conducted in MEGA11.

Table S4. Differences in alfalfa growth and stress parameters among treatments in the Inoculation*Salinity interaction with a 95% confidence interval. Results followed by the same letter are not significantly different according to Fisher's Least Significant Difference (LSD) post hoc test.

Treatment	Sample ID	Germination		Root Lenght		Shoot Lenght		Chlorophylls		Proline	
Inoculation-No*Salinity-0	A	95.3	a b	2.67	e	3.17	e	9.58	j	1.25	i
Inoculation-No*Salinity-100	B	88.0	d e	3.80	a b c	4.30	a b c	12.93	d e	3.13	b
Inoculation-No*Salinity-200	C	75.0	k	3.17	c d e	3.67	c d e	10.36	i	3.54	A
Inoculation-No*Salinity-300	D	68.0	l	2.83	d e	3.33	d e	6.85	k	3.45	A
Inoculation-No*Salinity-50	E	91.7	c	3.35	b c d e	3.85	b c d e	13.88	b c	3.52	A
Inoculation-Consortium 1*Salinity-0	F1	97.0	a	3.67	a b c	4.17	a b c	13.53	b c d	1.83	g h
Inoculation-Consortium 2*Salinity-0	F2	88.7	d	3.30	b c d e	3.80	b c d e	12.49	e f	1.68	h
Inoculation-Consortium 3*Salinity-0	F3	86.0	e f g	3.15	c d e	3.65	c d e	12.16	f g	1.64	h
Inoculation-Consortium 1*Salinity-50	G1	95.3	a b	3.75	a b c	4.25	a b c	15.33	a	2.09	f g
Inoculation-Consortium 2*Salinity-50	G2	87.7	d e	3.30	b c d e	3.80	b c d e	14.22	b	1.90	g h
Inoculation-Consortium 3*Salinity-50	G3	84.3	g h	3.20	b c d e	3.70	b c d e	13.06	d e	1.83	g h
Inoculation-Consortium 1*Salinity-100	H1	93.3	b c	4.33	a	4.83	a	13.27	c d	2.61	c
Inoculation-Consortium 2*Salinity-100	H2	85.3	f g	3.93	a b c	4.43	a b c	10.91	h i	2.56	c d
Inoculation-Consortium 3*Salinity-100	H3	82.3	h	3.77	a b c	4.27	a b c	10.54	i	2.48	c d e
Inoculation-Consortium 1*Salinity-200	I1	87.3	d e f	4.00	a b	4.50	a b	11.50	g h	2.55	c d
Inoculation-Consortium 2*Salinity-200	I2	79.7	i	3.63	a b c d	4.13	a b c d	10.56	i	2.44	c d e
Inoculation-Consortium 3*Salinity-200	I3	77.3	j	3.48	b c d e	3.98	b c d e	10.52	i	2.36	c d e f
Inoculation-Consortium 1*Salinity-300	J1	82.3	h	3.58	a b c d	4.08	a b c d	12.99	d e	2.50	c d e
Inoculation-Consortium 2*Salinity-300	J2	75.0	k	3.20	b c d e	3.70	b c d e	11.91	f g	2.29	d e f
Inoculation-Consortium 3*Salinity-300	J3	73.7	k	3.13	c d e	3.63	c d e	11.66	g	2.25	e f
LSD-value		2.3		0.83		0.83		0.71		0.27	

Table S5. Changes in *Triticum durum* growth and stress parameters from uninoculated to inoculated plants at the same salinity level.

Salinity	0 mM	250.0 mM	500.0 mM
**Root Length**	+ 110.8	+ 91.3	+ 38.9
**Shoot Length**	+ 71.4	+ 59.1	+ 112.0
**Chlorophyll a**	+ 226.1	+ 119.9	+ 17.9
**Chlorophyll b**	+ 216.7	+ 373.5	-46.4
**Chlorophyll tot**	+ 223.3	+ 208.2	-15.8
**Chl a / Chl b**	+ 3.4	-56.7	+ 116.7
**DPPH**	-18.4	-21.9	-14.0
**Proline**	+ 45.7	+ 19.9	+ 21.9
